# Correction to: Elf3 drives *β*-catenin transactivation and associates with poor prognosis in colorectal cancer

**DOI:** 10.1038/s41419-025-07972-8

**Published:** 2025-10-28

**Authors:** J.-L. Wang, Z.-F. Chen, H.-M. Chen, M.-Y. Wang, X. Kong, Y.-C. Wang, T.-T. Sun, J. Hong, W. Zou, J. Xu, J.-Y. Fang

**Affiliations:** 1 State Key Laboratory of Oncogene and Related Genes, Key Laboratory of Gastroenterology & Hepatology, Ministry of Health, Department of Gastroenterology and Hepatology, Ren-Ji Hospital, School of Medicine, Shanghai Jiao-Tong University, Shanghai Cancer Institute, Shanghai Institute of Digestive Disease, 145 Middle Shandong Road, Shanghai, China; 2 Department of Surgery, University of Michigan, Ann Arbor, MI USA

Correction to: *Cell Death & Disease* 10.1038/cddis.2014.206, published online 29 May 2014

Upon re-examination, we identified that we had made an unintentional and naive mistake, with a mix-up of pictures in the control group of Figure 5E. This error does not affect the main conclusions of the study.

**Original Figure** 
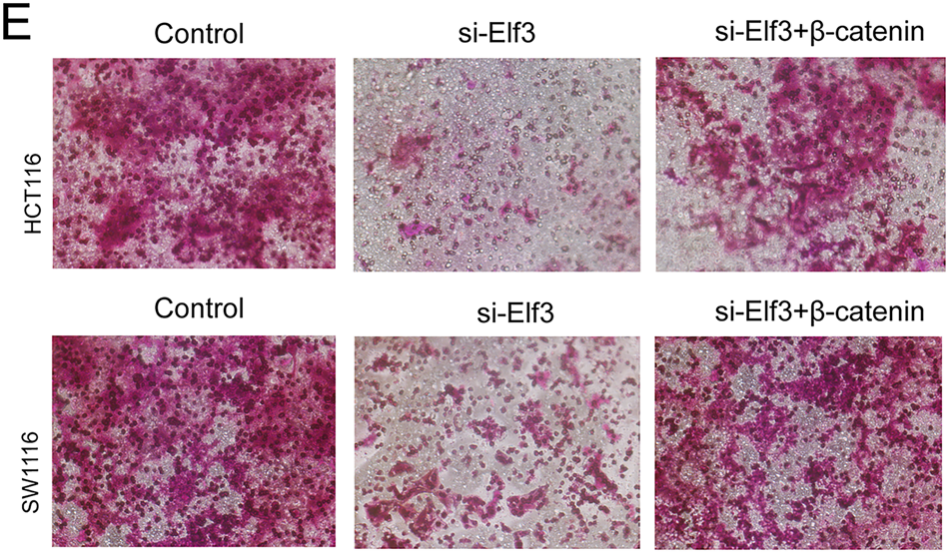


**Corrected Figure** 
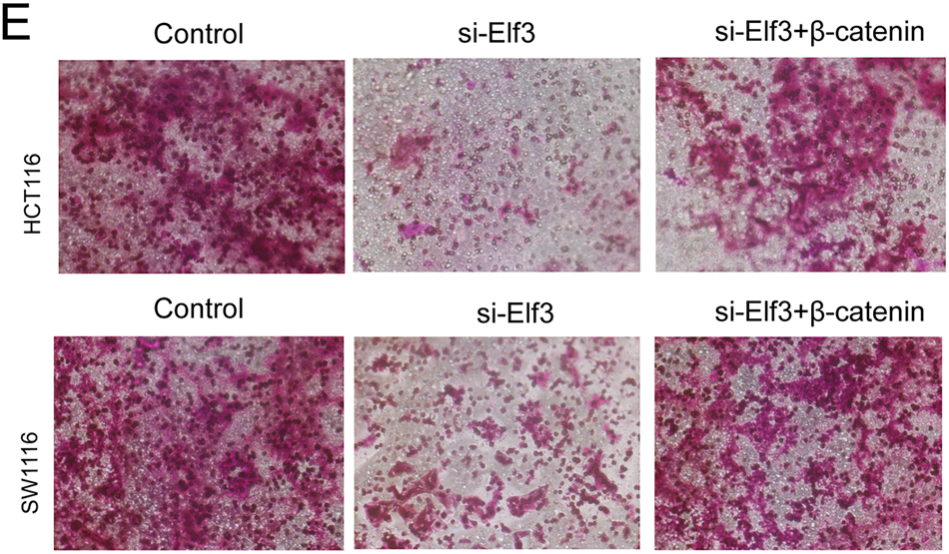


## Supplementary information

Revised_Supplemental figures

